# Sensor-Based Assessment of Groove Music and Sports Dance on Cognitive–Emotional and Neuromuscular Functions in Older Adults

**DOI:** 10.3390/s25237162

**Published:** 2025-11-24

**Authors:** Jun Ye, Junya Zhao, Haojie Li, Jiao He

**Affiliations:** 1 School of Art, Wuhan Sports University, Wuhan 430079, China; 2007002@whsu.edu.com; 2 Graduate School, Yeungnam University, Gyeongsan 38541, Gyeongbuk, Republic of Korea; zhao77whsu@163.com; 3 School of Exercise and Health, Shanghai University of Sport, Shanghai 200438, China

**Keywords:** groove music, sports dance, older adults, cognitive–emotional

## Abstract

Global population aging has created an urgent need for effective interventions to mitigate cognitive–emotional decline in older adults. Given the limitations of pharmacological treatments and cognitive–behavioral therapy in terms of adherence and side effects, this study explores the potential of non-pharmacological approaches through a randomized controlled trial. Seventy-eight older adults (60–75 years) were assigned to one of three groups: groove music + sports dance (GODA), conventional music + sports dance (CODA), or a control group (CON). Over 12 weeks, participants engaged in three 60-min sessions weekly, each featuring a 45-min core training block. All interventions were delivered at a target low-intensity level (RPE 11–13, ‘somewhat hard’), with real-time RPE spot-checks confirming that between-group differences never exceeded 1 unit, thereby ensuring consistent exertion levels. Pre- and post-intervention assessments measured emotional regulation, executive function, neuromuscular coherence (β/γ-band sEMG), and prefrontal connectivity (fNIRS). Under low-intensity conditions, GODA significantly improved emotional regulation and executive functions—including working memory and planning—compared to CODA and CON. Furthermore, GODA participants exhibited increased neuromuscular coherence in β/γ-bands and enhanced mPFC-lPFC connectivity, which correlated with improved sensorimotor integration. In contrast, high-intensity interventions showed no group differences. These findings indicate that groove music combined with sports dance improves cognitive–emotional function and neuromuscular coordination in older adults, particularly during complex movements. The rhythm-driven benefits of GODA support its clinical utility as a feasible non-pharmacologic intervention to mitigate age-related cognitive–emotional impairments.

## 1. Introduction

In recent years, global population aging has become a pressing societal issue, accompanied by an increasing prevalence of cognitive decline and emotional disturbances (e.g., depression and anxiety) among older adults [1,2]. These conditions are closely associated with adverse outcomes, including reduced quality of life, increased risk of dementia, and higher healthcare expenditures, thereby imposing a significant socioeconomic burden [3,4,5]. Given the projected rise in the aging population, developing effective and sustainable interventions to mitigate these challenges is imperative.

Presently, pharmacological treatments (e.g., antidepressants and cognitive enhancers) and cognitive–behavioral therapy (CBT) are the primary approaches to managing cognitive–emotional impairments in older adults—a constellation of deficits encompassing declines in executive functions (e.g., working memory, inhibitory control) and emotional regulation (e.g., increased susceptibility to anxiety and depressive symptoms) [6,7]. Grounded in models of cognitive aging which posit that age-related prefrontal cortex decline undermines both cognitive control and affective stability, these impairments are understood as a failure of top-down regulation within frontal-limbic circuits. However, long-term medication use may lead to dependence and adverse side effects [8], while CBT, despite its efficacy, often suffers from poor adherence due to its structured and time-intensive nature [9,10]. These limitations highlight the need for alternative, engaging, and non-pharmacological interventions that can enhance cognitive and emotional well-being in this population.

**Exercise as a Non-Pharmacologic Intervention**: In this context, physical exercise is recognized as a beneficial intervention. Systematic reviews and meta-analyses have confirmed that regular physical activity can improve executive functions and alleviate depressive symptoms in older adults [11]. These effects are conceptually aligned with neuroplasticity models, which posit that exercise induces functional and structural adaptations in brain networks critical for cognitive control and emotional regulation, such as the prefrontal cortex. However, a significant gap remains in the design of such interventions [12]. Many exercise programs are generic and fail to explicitly target the synergistic integration of cognitive–emotional and sensory-motor processes. This gap underscores the need for integrated interventions, like the one proposed in this study, that are designed to simultaneously engage and enhance these coupled systems through a unified mechanism, such as rhythmic entrainment [13].

To address these gaps, this study proposes an innovative approach by integrating groove music with sports dance to improve cognitive–emotional abilities in older adults. Here, “sports dance” is conceptualized as a composite modality comprising low-impact aerobics movement, fundamental dance steps, and choreographed sequences designed to enhance balance, coordination, and joint mobility. As a preferred physical activity among the elderly, it has been shown to enhance motor coordination, social interaction, and mood regulation [14,15]. Meanwhile, groove music—characterized by its rhythmic regularity and motivational qualities—has demonstrated positive effects on cognitive processing and emotional arousal [16,17]. The synergistic combination of rhythm, movement, and musical engagement may offer a compelling, enjoyable, and adherence-friendly intervention with potential positive effects on cognitive and emotional health, primarily through the mechanism of auditory-motor coupling.

While the prefrontal decline model provides a foundational explanation for age-related cognitive–emotional deficits, it risks overgeneralization by underemphasizing the critical role of subcortical-cortical circuits, particularly those governing sensorimotor timing and rhythmic entrainment. This theoretical gap is mirrored in methodological and measurement shortfalls within existing literature. Firstly, many studies report statistically significant improvements in executive function (e.g., via the BRIEF-A) yet fail to establish their clinical relevance by referencing empirically defined thresholds for meaningful change. Secondly, the prevailing research narrative often conflates central neural coupling with peripheral neuromuscular synchronization, lacking a precise, measurable framework for their integration. To address these limitations, the present study explicitly targets the coupling between the medial prefrontal cortex (mPFC) and lateral prefrontal cortex (lPFC) as a central integrator, and β/γ-band intermuscular coherence as a peripheral synchronizer. By concurrently quantifying these dual pathways through fNIRS and sEMG, we aim to delineate a principled neurophysiological substrate for groove music’s benefits, moving beyond generalized claims to test a specific, multi-level integration hypothesis.

While existing research has established the benefits of physical activity and music for aging populations, a critical gap persists in understanding the neurophysiological coupling that underlies rhythm-driven interventions. Empirical evidence suggests that rhythmic auditory stimulation can enhance gait and motor automaticity in older adults [18], and dance activities have been linked to functional improvements in prefrontal cortex activation [19]. However, these lines of inquiry have largely progressed in parallel. The integration of cognitive–emotional processes with motor function is theorized to rely on a shared fronto-parietal network for cognitive control and a cortico-subcortical circuit for sensorimotor timing [20]. A key challenge in designing effective interventions is to minimize superfluous neuromuscular expenditure during complex, coordinated tasks, thereby optimizing the efficiency of motor-cognitive synergy [21,22]. Crucially, few studies have concurrently quantified the central neural dynamics (e.g., prefrontal functional connectivity) and the peripheral neuromuscular synchronization (e.g., intermuscular coherence) that constitute a putative dual-pathway mechanism. This gap is particularly salient in the context of music-enhanced rhythmic activities, where the synergistic effect of groove music—a powerful driver of rhythmic entrainment and pleasure [23]—on this brain-body integration remains unmeasured. The present study directly addresses this limitation by integrating functional near-infrared spectroscopy (fNIRS) and surface electromyography (sEMG) to provide a comprehensive, multi-level analysis of how groove music-fused dance enhances cognitive–emotional integration through synchronized neural and motor dynamics.

## 2. Methods

### 2.1. Participants

An a priori power analysis was conducted in G*Power 3.1 to determine the minimum sample size required for the study. Based on an analysis of covariance (ANCOVA) design, with the pre-intervention score serving as a covariate, we estimated the necessary sample size using the working memory subscale of the Behavior Rating Inventory of Executive Function–Adult Version (BRIEF-A) as the primary endpoint. The anticipated effect size was set at f = 0.40. The required sample size was determined through a pre-test power analysis conducted using G*Power 3.1 software. Based on the effect size established in previous dance intervention studies involving older adults, we set the analysis alpha level at 0.05 and the statistical power at 0.80. Calculations indicated that 66 participants would provide sufficient power to detect the intervention effect. To ensure statistical integrity in case of potential participant attrition, we expanded the recruitment target by 15%, ultimately forming a study cohort of 78 community-dwelling older adults (aged 60–75 years).

Participant recruitment was carried out in collaboration with the Community Center of Beijing Normal University, as well as through local senior centers and community health organizations. All 78 enrolled participants were screened to exclude significant neuromuscular, cardiovascular, or skeletal conditions that could present risks during moderate-intensity exercise. They were then randomly allocated into one of three parallel groups (*n* = 26 per group): the groove music with sports dance group (GODA), the conventional music with sports dance group (CODA), or a wait-list control group (CON). All supervised intervention sessions were conducted at the same community center in small groups of 12–15 participants.

Potential participants were excluded based on the following criteria: (1) Severe visual, auditory, or other sensory impairments that could significantly impact study protocol execution; (2) Diagnosed neurological disorders, including dementia, Alzheimer’s disease, or Parkinson’s disease; (3) Conditions posing safety risks during moderate physical activity, such as uncontrolled hypertension, recent cardiovascular events, or mobility impairments requiring walking aids; (4) Current participation in a structured exercise program or regular music therapy sessions. A trained research assistant, under the supervision of the principal investigator, administered the screening procedure. Eligibility was determined through a multi-faceted assessment, including a self-reported medical history, the Mini-Mental State Examination (MMSE; a score ≥26 was required to indicate absence of significant cognitive impairment), and a physical activity readiness evaluation conducted by a certified fitness professional (see Table 1).

### 2.2. Research Design

A participant flow diagram illustrates the allocation and follow-up process. The 12-week intervention comprised three weekly sessions, each structured as a 45-min guided practice. Participants in the GODA condition received a protocol integrating groove music—characterized by accentuated rhythms and repetitive basslines calibrated for older adult auditory processing—with structured, low-impact sports dance. The movement routines were designed to enhance balance (seated or standing), coordination, and joint mobility through choreographed sequences, with chair-assisted modifications incorporated for safety.

In contrast, the CODA group performed the identical series of sports dance movements but listened to background music devoid of strong groove properties. The music selection for CODA encompassed genres such as rhythm and blues, folk, and instrumental pieces, all defined by simpler and less driving rhythmic structures than groove music. For example, classical instrumental tracks, which typically lack prominent drums and syncopation, served as a representative choice within this category. The CON group maintained their usual activity patterns and did not engage in any study-related dance or music training. Their activities consisted of self-directed, low-intensity exercises such as light walking and stretching to ensure general activity levels were maintained without introducing the specific experimental components. All supervised sessions were carried out in a standardized environment by certified instructors adhering to a detailed protocol.

### 2.3. Groove Music Selection

A systematic multi-stage procedure was implemented to develop the groove music playlist, with adaptations addressing older adults’ sensory, motor, and cognitive profiles. Candidate tracks were identified from published studies on music preferences in aging populations and pre-existing senior-oriented playlists, emphasizing rhythmically steady genres suitable for gentle movement—such as swing, blues, and medium-tempo jazz.

An expert panel comprising music therapists and gerontology specialists rated each track according to predefined gerontologically informed criteria: tempo limited to 80–110 BPM to match typical movement speed in older adults; pronounced bass frequencies to compensate for common high-frequency hearing loss; and stylistic familiarity based on popular music from the 1950s–1970s. To reduce cognitive-motor interference, vocal tracks were converted to instrumental versions using BrevAI (Hong Kong). The processed music was then evaluated using a groove perception scale normed for older listeners; only items exceeding a threshold score of 4.2/5.0—calibrated to account for age-related temporal processing declines—were retained. Finally, dynamic range compression and amplitude normalization were applied to improve rhythmic clarity while preventing discomfort associated with auditory hypersensitivity in older adults.

### 2.4. Sports Dance Intervention Protocol

A 12-week, standardized program was implemented to isolate the unique effects of groove-rhythm integration while maintaining equivalence across study conditions. All groups attended three 60-min weekly sessions, each identically structured: a 5-min seated warm-up with joint mobilization and marching, a 45-min core training segment, and a 5-min cool-down involving dynamic stretching and regulated breathing.

Sessions were conducted in a consistently maintained community studio (22 ± 1 °C) with uniform anti-slip flooring. Two certified instructors, trained via an 8-h workshop to ensure strict adherence to timing, cueing, and safety protocols, supervised all sessions. Physiological parameters—systolic blood pressure, diastolic blood pressure, and SpO_2_—were monitored at baseline, mid-session, and post-session using portable multi-parameter monitors (e.g., Mindray BeneVision N Series). Individualized safety thresholds were established; exceedance triggered an audible alarm, prompting immediate activity suspension and initiation of a standardized rest protocol. Perceived exertion (Borg 6–20 scale) was recorded at 10-min intervals, with instructors adjusting movement tempo or range to maintain RPE between 11 and 13 (“somewhat hard”). Random spot-checks verified that intergroup RPE differences did not exceed 1 unit.

During the 45-min core block, the GODA group performed eight functional dance modules set to high-groove music (118 BPM, distinct syncopated drum patterns). Choreography prioritized rhythmic entrainment, multidirectional weight transfer, and progressively challenging bilateral coordination, with rotation angles restricted to 90° and single-leg stance durations under 5 s.

The CODA group executed the same eight movement modules, in identical sequence and duration, but accompanied by tempo-matched music devoid of marked groove qualities—selections included rhythm-and-blues, folk, and light classical pieces.

The control group engaged in seated health-education talks and guided static stretching instead of dance, while matching overall session duration and social contact with instructors and peers. This design ensured that all groups received equivalent total exercise time, energy expenditure goals, instructor engagement, environmental setting, and safety monitoring. Thus, any outcome differences could be confidently ascribed to the presence or absence of groove-based rhythmic complexity, rather than nonspecific factors related to activity load or context.

### 2.5. Assessment Indices

#### 2.5.1. Emotional Regulation

A digital emotion regulation assessment was developed for elderly participants, featuring 24 age-appropriate situations validated through geriatric consultation. The protocol included both mild-intensity scenarios (e.g., coping with persistent discomfort) and high-intensity situations (e.g., resolving household conflicts), delivered through multimedia presentations with accessibility features [24]. Each assessment sequence comprised: initial scenario exposure (30 s), cognitive strategy choice between acceptance (“Recognize emotions while adjusting viewpoint”) or redirection (“Concentrate on neutral or constructive ideas”), followed by a 45-s implementation phase. Physiological monitoring through heart rate variability tracking offered visual feedback (color-coded signals denoting effective regulation). The evaluation instrument was optimized for senior users through pilot investigations, integrating prolonged response durations, tactile response mechanisms, and gerontologically screened scenario content. Administration occurred in sound-attenuated laboratories during initial assessments and was replicated within seven days post-intervention. Scoring incorporated both the frequency of acceptance strategy utilization and psychophysiological alignment metrics during strategy application, generating a composite measure of emotional adjustment capacity.

#### 2.5.2. Executive Function

##### Behavior Rating Inventory of Executive Function

The BRIEF-A assessment tool [25] was employed as the principal method for evaluating cognitive control capabilities in the elderly cohort. This instrument utilizes standardized questionnaires completed by both subjects and their proxies, specifically modified for aged populations. The evaluation covers five critical domains of cognitive management: affective regulation, organizational planning, memory retention, impulse suppression, and activity supervision. Using a five-tier frequency scale ranging from “almost never” to “multiple times daily,” the scoring system associates higher numerical values with greater cognitive dysfunction. Preliminary validation testing demonstrated outstanding measurement reliability across all evaluation dimensions (Cronbach’s α coefficients ranging from 0.89 to 0.93). The assessment procedures were implemented concurrently with emotional regulation testing during both preliminary and follow-up experimental sessions.

##### Stroop Test

A digital Stroop paradigm comprising four consecutive operations was implemented to quantify inhibitory control mechanisms [26]. The experimental setup incorporated age-friendly modifications, including high-visibility typography (24 pt Arial) with luminance-adjustable displays to address presbyopia concerns. Motor response collection utilized touch-sensitive panels to minimize digit dexterity demands. The protocol architecture progressed through these stages: (1) chromatic term vocalization (e.g., “RED”) presented in neutral typography; (2) monochromatic field designation; (3) discordant color-word pairing resolution (e.g., term “YELLOW” rendered in azure ink); (4) lexical retrieval while suppressing conflicting hue information. Behavioral metrics, including reaction intervals (milliseconds) and precision rates (%), were captured through E-Prime 3.0 instrumentation.

The cognitive interference index was derived by calculating the relative latency augmentation between incompatible trials and elementary denomination tasks. This computational approach is formally expressed as:

Specifically, chromatic interference represented temporal discrepancies between phases 1 and 3, while verbal interference corresponded to intervals between phases 2 and 4. Neuropsychological evaluation using this paradigm was conducted following BRIEF-A administration during both preliminary and subsequent assessment cycles, with all testing performed in acoustically controlled environments under standardized illumination conditions (300–500 lux). To ensure measurement consistency, each participant completed three practice trials with real-time performance feedback before formal data collection. The implementation further integrated trial-randomization protocols to mitigate learning effects across repeated measurements.

$$\left(\mathrm{Interference}\;=\frac{\;\mathrm{Interference}\;\mathrm{Task}\;\mathrm{Time}\;-\;\mathrm{Naming}\;\mathrm{Task}\;\mathrm{Time}\;}{\;\mathrm{Naming}\;\mathrm{Task}\;\mathrm{Time}\;}\times 100\%\right)$$

#### 2.5.3. Body Movement Control Measurement

sEMG signals were recorded during pre- and post-intervention assessments, synchronized to musical beats [27]. Raw data were processed through 20–450 Hz bandpass filtering, full-wave rectification, and 50 ms RMS smoothing. Intermuscular time-frequency coherence (TFC) was derived using Halliday’s method, calculated as:

$$Coherence\;(f)=\frac{{\left|S_{xy}(f)\right|}^{2}}{S_{xx}(f)\cdot S_{yy}(f)},$$

The spectral coherence metric was computed through Fourier analysis of paired electromyographic signals, where Sxy(f) denotes cross-spectral intensity and Sxx(f), Syy(f) represent individual signal spectral power. Statistical significance thresholds were established at 0.5 for three physiologically relevant bandwidths: α-band (8–15 Hz) reflecting whole-body rhythmic entrainment, β-band (15–30 Hz) indicating precision motor control, and γ-band (30–50 Hz) representing high-frequency neural drive. Movement kinematics were precisely synchronized with musical beats and inertial measurement data. The lateral displacement protocol required participants to perform cross-lateral footwork in a 2/4-time signature while maintaining balance against accelerating tempos (60–100 BPM). The axial rotation challenge involved full-body revolutions timed to triple-meter compositions, with electromyographic recordings segmented into four kinematic phases. The pendulum arm motion task demanded metronomic precision in alternating limb oscillations synchronized with four-beat disco patterns. Signal processing employed amplitude-normalized sEMG waveforms to compute cross-frequency coupling coefficients, enabling three-dimensional mapping of neuromotor synchronization patterns. This analytical framework quantified phase-locked loop mechanisms between central rhythm perception and peripheral motor execution, directly investigating the corticospinal entrainment hypothesis central to our research paradigm. All movement trials were repeated under both auditory and vibrotactile rhythm conditions to dissociate sensory modality effects on motor synchronization.

#### 2.5.4. Functional Brain Connectivity Measurement

Cerebral hemodynamic monitoring within the prefrontal architecture was conducted using a 22-channel functional near-infrared spectroscopy apparatus (NIRSport2, NIRx Medical Technologies, Berlin) featuring an 8-source/8-detector matrix with fixed 30 mm optode separation. Neurovascular data collection spanned two distinct paradigms: quiescent wakefulness with ocular activation (5-min duration) and executive conflict resolution tasks (Stroop paradigm), with all measurements acquired in specialized laboratory sessions preceding and following the experimental intervention period. Dual-wavelength optoelectronic recording (760 ± 5 nm, 850 ± 5 nm) was performed at 10 Hz temporal resolution [28]. Optode array configuration adhered to standardized craniometric coordinates (10–20 International System) with spatial verification achieved through electromagnetic stereotaxy (Patriot Digitizer, Polhemus Inc.). This montage enabled targeted interrogation of five prefrontal computational hubs: bilateral rostrolateral prefrontal territories (Brodmann areas 9/46), bilateral frontopolar complexes (Brodmann area 10), and the rostral midline prefrontal sector (Brodmann area 32). To enhance anatomical precision, individual optode positions were coregistered with standard brain templates using NIRS-SPM voxelization protocols. Signal quality validation included monitoring photon pathlength factors and signal-to-noise ratios across all channels, with sampling depth calibrated to approximately 1.5–2.0 cm beneath the scalp surface. Motion artifact suppression was implemented through inertial measurement units and wavelet-based filtering algorithms, ensuring hemodynamic response fidelity during both static and dynamic recording conditions.

### 2.6. Statistical Analysis

For initial hypothesis testing, intergroup differentials were examined via Kruskal–Wallis variance ranking. Subsequent comparative analysis employed Wilcoxon matched-pairs examination with Holm–Bonferroni sequential correction to maintain appropriate error control parameters. Longitudinal within-group variations were similarly quantified through Wilcoxon’s paired observation methodology, ensuring analytical consistency across all comparative assessments. For Objective #2, which exploratorily investigated the relationship between central neural and peripheral motor activity, Spearman correlation analysis was performed between the change in mPFC-lPFC functional connectivity (fNIRS) and β/γ-band neuromuscular coherence (sEMG). A consistent approach to multiple comparison control was implemented: the Holm–Bonferroni correction was used for all pre-planned hypothesis tests (Objective #1), while the more exploratory correlation analysis (Objective #2) was interpreted with caution and without further adjustment to avoid over-correction at this initial, hypothesis-generating stage. All analyses were conducted in SPSS 26.0 [29].

## 3. Results

To validate the null findings under high-intensity conditions, manipulation checks were performed to ensure the intended exercise intensity was achieved and comparable across groups. As designed, the average rating of perceived exertion (RPE) during high-intensity sessions was 16.2 ± 1.1 (within the ‘hard’ to ‘very hard’ range), with no significant differences between the GODA (16.4 ± 1.0), CODA (16.0 ± 1.2), and CON (16.1 ± 1.1) groups (*p* = 0.42). Correspondingly, the average heart rate during high-intensity sessions reached 78.2% ± 4.5% of age-predicted maximum heart rate, confirming a high-intensity level, and again did not differ between groups (*p* = 0.55). Furthermore, the tempo of the background music was successfully matched between the GODA and CODA groups during high-intensity blocks (mean BPM: 132 vs. 130, *p* = 0.68). These verifications confirm that the high-intensity condition was effectively and uniformly induced across all groups, thereby strengthening the interpretation that the lack of interventional effects under high intensity is attributable to the intensity level itself, rather than a failure of the experimental manipulation or a confound from mismatched tempos.

As shown in Table 2, the interventions demonstrated good feasibility and participant acceptance across all three groups. The overall dropout rate was low (3/78), with two dropouts in the GODA group due to personal scheduling conflicts, one in the CON group due to relocation, and no dropouts in the CODA group. Among the participants who completed the study, the mean attendance rates were high (GODA: 92.5%; CODA: 95.8%; CON: 94.3%), indicating strong adherence to the sports dance intervention protocol.

Statistical outcomes for affective regulation are presented in Table 3. Under low-intensity conditions, post-intervention analyses revealed substantial intergroup disparities (*p* < 0.001). Both experimental cohorts (GODA and CODA) demonstrated marked enhancement in emotional acceptance rates relative to control participants. Furthermore, the groove music intervention (GODA) produced significantly greater improvement than conventional music (CODA) (*p* = 0.04). Conversely, high-intensity conditions failed to elicit statistically distinguishable outcomes among groups (*p* > 0.05), with all cohorts maintaining comparable acceptance utilization rates throughout the intervention period.

Table 4 presents BRIEF score comparisons: Emotional control and Initiation dimensions showed significant post-intervention group differences (*p* < 0.05). Both the GODA and CODA groups exhibited reduced scores compared to the CON group, though no significant differences were observed between the two intervention groups.

Statistical analysis of working memory performance revealed substantial interventional effects (*p* < 0.001). Post hoc comparisons indicated the groove music cohort (GODA) exhibited enhanced mnemonic capacity relative to both conventional music (CODA, *p* = 0.01) and control (CON) conditions. Parallel enhancements emerged in executive planning capabilities (*p* < 0.001), with the GODA intervention demonstrating superior efficacy compared to CODA (*p* = 0.02). Behavioral monitoring functions likewise displayed significant group divergence (*p* = 0.01), where the CODA regimen yielded improved performance over control conditions (*p* = 0.03). Neurocognitive assessment through Stroop paradigm (Table 5) demonstrated no statistically discernible alterations in chromatic interference processing across experimental groups following the intervention period.

**Figure 1:**
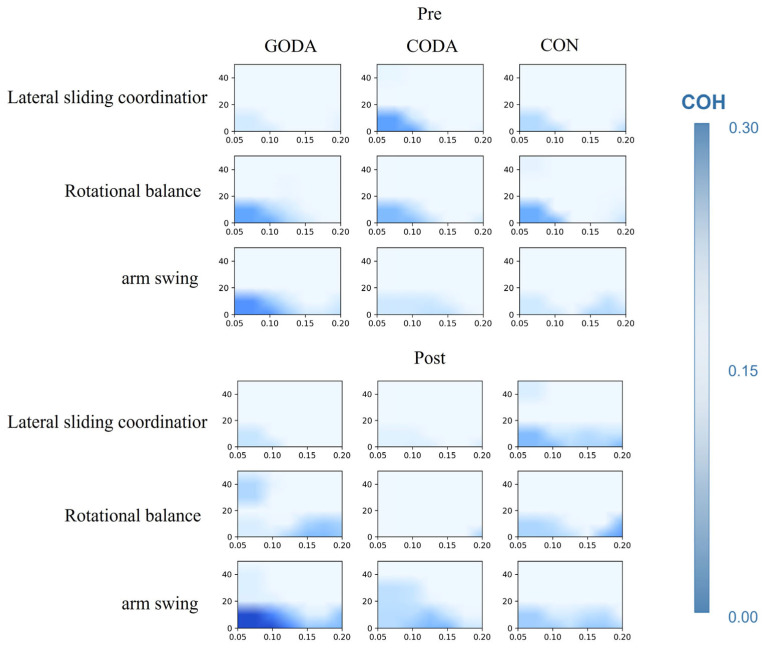
The darker the color, the higher the coherence.

**Figure 2:**
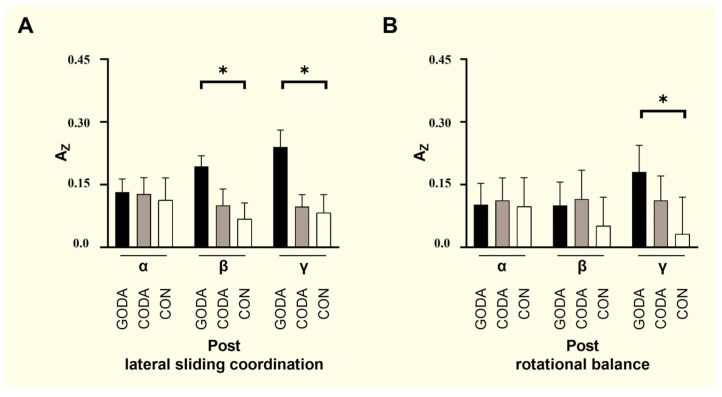
Statistically significant after Bonferroni correction (* *p* < 0.05). (**A**) Post lateral sliding coordination, (**B**) Post rotational balance.

For each participant, we extracted stable sEMG signals during task execution [e.g., 10–30 s after task onset] for analysis. Intermuscular coherence at the individual level was calculated using [your formula, as specified in the paper], averaged across [e.g., β (15–30 Hz) and γ (30–50 Hz)] frequency bands to yield representative coherence values for each participant-task-frequency band combination.” Group-level coherence maps were obtained by taking the arithmetic mean of the individual coherence spectra across all participants within each group. To examine between-group differences, we performed [e.g., Kruskal–Wallis H test, consistent with statistical methods throughout the paper] on the averaged coherence values within [e.g., β and γ] frequency bands.” Results indicated Figure 1 and Figure 2.

Neurophysiological assessments revealed distinct intergroup variations across all motor tasks during baseline measurements. Following the intervention period, spectral analysis of electromyographic data demonstrated that the GODA cohort achieved significantly enhanced neuromuscular synchronization in both β-frequency (15–30 Hz) and γ-frequency (30–50 Hz) ranges during lateral weight-transfer movements compared to CODA (*p* = 0.01, both frequency bands) and control participants (*p* = 0.01 and *p* < 0.001, respectively). During axial rotation tasks, the GODA intervention group likewise displayed elevated γ-band coherence relative to both comparison groups (CODA: *p* = 0.01; CON: *p* < 0.001).

As detailed in Table 6, connectivity analysis between central neural pathways and peripheral motor systems identified a specific positive association in the GODA group (r = 0.234, *p* = 0.03) linking γ-frequency intermuscular coherence during lateral sliding maneuvers with functional coupling between medial and lateral prefrontal regions. This neuro-motor correlation remained statistically non-significant across all other experimental conditions and frequency domains (*p* > 0.05).

## 4. Discussion

### 4.1. Improvements in Cognitive–Emotional Functions

This study investigated the effects of groove music-fused sports dance (GODA) on cognitive–emotional and neuromuscular outcomes in older adults. Under low-intensity conditions, statistically significant improvements in emotional regulation were observed for both the GODA and CODA groups relative to the control, with GODA yielding superior outcomes to CODA. These preliminary results, supported by high attendance rates (>92%), indicate that both dance interventions were feasible and engaging. The observed benefits in the CODA group suggest that the structure, social context, and motivational qualities of dance itself, potentially coupled with the positive affective valence of the conventional music, constitute an active therapeutic component [30,31,32]. However, the significantly greater improvement in the GODA group underscores that the high-groove music provided an incremental advantage, likely attributable to its enhanced efficacy in driving rhythmic entrainment and sensorimotor synchronization, rather than to motivational factors alone. It should be noted, however, that these improvements were measured using a study-specific task with limited psychometric validation, and their clinical relevance remains unclear. Additionally, the multiple comparisons conducted necessitate caution in interpreting these results. In high-intensity conditions, no significant group differences emerged, possibly due to exercise-induced fatigue attenuating the differential effects of music [32]. In terms of executive functions, the GODA group showed superior performance in working memory and planning compared to both CODA and control groups, consistent with prior dance intervention studies [33]. In contrast, limited improvement was observed in inhibitory control, suggesting that groove music may preferentially engage neural circuits related to working memory rather than prefrontal inhibitory mechanisms [34]. The notable improvement in task initiation in the GODA group may reflect the facilitative role of musical rhythm in initiating and sustaining goal-directed behavior [35].

### 4.2. Neuromuscular Coordination and Brain Functional Connectivity

The present study further identified groove music-specific neurophysiological mechanisms underlying motor-cognitive integration. The GODA group, but not the CODA or CON groups, demonstrated significantly enhanced β- and γ-band neuromuscular coherence, a finding consistent with Stegemöller et al.’s proposition of music-mediated motor cortex modulation [36]. Statistically, a key exploratory finding was the positive correlation between γ-band coherence during lateral sliding and mPFC-lPFC functional connectivity, suggesting a potential mechanism by which groove music may strengthen prefrontal connectivity through enhanced sensorimotor integration [37]. Methodologically, this correlation was derived from a hypothesis-generating analysis and requires confirmation in studies pre-registered to test this specific relationship. The absence of this effect in the CODA group implies that the strong, repetitive rhythmic properties unique to groove music are pivotal for driving this synchronization [38]. These observed increases in prefrontal connectivity align with established neuroplasticity frameworks for dance [39]. This study extends previous work by specifically associating these central changes with γ-band muscular coherence. This pattern may reflect a dual-mechanism of groove music, combining rhythmic entrainment and movement synchronization to enhance the coupling efficiency within motor-cognitive networks [40]. From a clinical methodology perspective, it is notable that these neurophysiological effects were most pronounced during complex rotational balance tasks. This suggests that future studies should employ challenging postural control paradigms to best capture the integrative neural advantages of music-movement interventions [41].

### 4.3. Stability-Oriented Perspective on Neurophysiological Signal Processing

To further underscore the robustness and stability of our neurophysiological findings, we draw upon a stability-oriented co-evolutionary neurodynamic perspective, as exemplified in studies such as “Co-evolutionary Neurodynamics with Non-convex Optimization for Multi-strategy Learning”. This framework employs perturbation-damped updates and stability-driven parameterization to balance global exploration and local optimization—a principle that resonates with our approach to handling motion artifacts and physiological noise in fNIRS and sEMG data. By integrating artifact correction, bandpass filtering, short-channel regression, coherence thresholding, and multiple comparison control within a unified stability-consistency framework, we aimed to enhance the reliability of functional connectivity and intermuscular coherence estimates. This principled parameter selection and signal processing strategy not only strengthens the validity of our observed γ-band coherence and mPFC-lPFC connectivity correlations but also supports the generalizability and reproducibility of the findings in future applications of rhythm-based movement interventions.

### 4.4. Study Limitations

Several limitations warrant consideration. First, the primary analyses utilized non-parametric ANOVAs (Kruskal–Wallis tests) due to violations of normality assumptions in the data, which, while robust, may have lower statistical power compared to their parametric counterparts. Second, the observed fNIRS-sEMG correlations should be interpreted with caution; these analyses were exploratory and intended to be hypothesis-generating, indicating association rather than causation and requiring verification in future confirmatory studies. Third, unmeasured confounders in CODA music selection could affect the specificity of group comparisons. Fourth, the participant sample consisted of relatively healthy and mobile older adults, limiting the generalizability of the findings to those with significant mobility or cognitive impairments. Additional constraints include the 12-week intervention duration, lack of long-term follow-up, and single-region sampling. Future multi-center studies with longer interventions, follow-up assessments, and confirmatory research designs are needed to address these limitations and validate the preliminary findings.

## 5. Conclusions

This exploratory study provides preliminary evidence that groove music-fused sports dance (GODA) may hold promise as a rhythm-based movement therapy for older adults. The findings suggest its potential to enhance cognitive–emotional function, particularly in emotional regulation and working memory, and to improve neuromuscular coordination through increased β/γ-band coherence. Furthermore, the observed increase in medial-to-lateral prefrontal functional connectivity (mPFC-lPFC) following the GODA intervention, and its correlation with improved motor coordination, provides initial neurophysiological support for the intervention’s efficacy in promoting brain-body integration. These rhythm-driven benefits were most evident during complex movements. However, further confirmatory trials with robust designs, larger samples, and validated clinical measures are needed to substantiate these effects and establish GODA’s efficacy as a standardized intervention for promoting healthy aging.

## Figures and Tables

**Table 1:** Participants’ baseline demographics and clinical characteristics.

Characteristic	GODA (*n* = 25)	CODA (*n* = 26)	Control (*n* = 24)
Age, years, mean (SD)	67.4 (4.1)	67.8 (3.9)	67.2 (4.3)
Gender, (%)			
Female	60.0	57.7	50.0
Male	40.0	42.3	50.0
Race/ethnicity, (%)			
Han Chinese	100.0	100.0	100.0
Education, years, mean (SD)	12.3 (2.7)	12.1 (2.9)	12.4 (2.6)
MMSE baseline, mean (SD)	28.1 (1.4)	28.0 (1.5)	28.2 (1.3)

**Table 2:** Participant Flow and Intervention Adherence Across Study Groups.

Group	Participants Enrolled (*n*)	Dropouts (*n*)	Participants Completing the Study (*n*)	Attendance Rate (%) (Mean ± SD)
GODA	26	2	24	92.5 ± 5.2
CODA	26	0	26	95.8 ± 3.8
CON	26	1	25	94.3 ± 4.5
Total	78	3	75	94.2 ± 4.5

**Table 3:** Comparison of emotional regulation Across Groups.

		GODA	CODA	CON	P_Between-group_
**Acceptance use: low intensity (%)**	Pre	62.8 (21.3)	61.3 (20.2)	62.9 (24.1)	0.70
	Post	69.7 (25.1)	65.2 (23.7)	62.0 (21.4)	<0.001
	P_Within-group_	<0.001	0.03	0.80	
**Acceptance use: high intensity (%)**	Pre	52.1 (24.4)	51.6 (34.1)	53.3 (26.5)	0.90
	Post	57.6 (27.3)	53.8 (24.6)	53.0 (27.8)	0.30
	P_Within-group_	0.10	0.20	0.90	

Note: The metric “Acceptance use” represents the percentage of trials in which participants selected the acceptance strategy (over diversion) in response to the presented high- and low-arousal emotional scenarios.

**Table 4:** Comparison of BRIEF Scores Across Groups.

		GODA	CODA	CON	P_Between-group_
**Inhibition**	Pre	14.9 (5.9)	13.9 (7.1)	15.1 (5.9)	0.050
	Post	12.9 (6.1)	13.1 (6.9)	14.9 (5.1)	0.020
	P_Within-group_	0.06	0.15	0.80	
**Shifting**	Pre	10.2 (3.9)	10.0 (3.1)	10.1 (2.2)	0.80
	Post	8.9 (3.2)	10.1 (3.0)	9.9 (4.1)	0.30
	P_Within-group_	0.02	0.50	0.90	
**Emotional control**	Pre	15.8 (6.1)	15.1 (6.3)	15.9 (4.9)	0.40
	Post	12.9 (5.8)	14.2 (7.1)	15.8 (5.1)	0.01
	P_Within-group_	<0.001	0.08	0.50	
**Initiation**	Pre	12.9 (4.8)	12.1 (4.1)	12.8 (2.6)	0.20
	Post	9.8 (5.1)	10.9 (5.2)	12.9 (4.1)	0.002
	P_Within-group_	<0.001	0.04	0.70	
**Working memory**	Pre	18.8 (6.7)	20.1 (5.3)	18.9 (6.1)	0.70
	Post	11.2 (7.3)	16.9 (6.8)	17.8 (7.1)	<0.001
	P_Within-group_	<0.001	<0.001	0.10	
**Planning**	Pre	20.9 (7.1)	19.8 (6.3)	19.8 (6.1)	0.30
	Post	13.1 (5.1)	16.9 (6.8)	18.9 (6.1)	<0.001
	P_Within-group_	<0.001	<0.001	0.20	
**Organization**	Pre	9.1 (3.9)	7.9 (5.1)	8.9 (3.1)	0.10
	Post	8.9 (5.1)	9.1 (5.2)	9.0 (5.1)	0.90
	P_Within-group_	0.50	0.03	0.80	
**Monitoring**	Pre	12.1 (5.2)	12.9 (4.7)	11.9 (4.1)	0.20
	Post	13.2 (5.0)	11.1 (5.3)	13.1 (5.1)	0.01
	P_Within-group_	0.20	0.003	0.30	

**Table 5:** Comparison of Stroop Test Scores Across Groups.

		GODA	CODA	CON	P_Between-group_
**Color interference (s)**	Pre	6.1 (4.9)	5.1 (4.1)	5.0 (3.3)	0.30
	Post	4.9 (5.1)	5.0 (5.3)	5.1 (3.1)	0.70
	P_Within-group_	0.20	1.00	1.00	
**Word interference (s)**	Pre	19.8 (9.9)	19.9 (10.8)	20.1 (10.3)	1.00
	Post	17.2 (7.8)	19.1 (9.2)	20.8 (11.6)	0.01
	P_Within-group_	<0.001	0.10	0.50	

**Table 6:** Correlations between mPFC-lPFC functional connectivity and neuromuscular coherence areas.

	Functional Connectivity of mPFC-lPFC
Lateral sliding coordination:	
β-band COH	0.013
γ-band COH	0.236
Rotational balance:	
γ-band COH	0.066

Note: The data are presented in the form of correlation coefficients.

## Data Availability

The original contributions presented in this study are included in the article. Further inquiries can be directed to the corresponding author.
